# Alzheimer’s disease severity and its association with patient and caregiver quality of life in Japan: results of a community-based survey

**DOI:** 10.1186/s12877-018-0831-2

**Published:** 2018-06-14

**Authors:** William Montgomery, Amir Goren, Kristin Kahle-Wrobleski, Tomomi Nakamura, Kaname Ueda

**Affiliations:** 1 Global Patient Outcomes & Real World Evidence, Eli Lilly Australia, 112 Wharf Rd, West Ryde, NSW 2114 Australia; 2 Health Outcomes Practice, Kantar Health, 11 Madison Ave, Floor 12, New York, NY 10010 USA; 3 Global Patient Outcomes & Real World Evidence, Eli Lilly and Company, Lilly Corporate Center, Indianapolis, IN 46285 USA; 4 Medical Development Unit, Eli Lilly Japan K.K, 4-15-1, Akasaka, Minato-ku, Tokyo, 107-0052 Japan; 5 Health Outcomes, Health Technology Assessment, & Real World Evidence, Medical Development Unit, Eli Lilly Japan K.K, 5-1-28, Isogami-dori, chuou-ku, Kobe, 651-0086 Japan

**Keywords:** Alzheimer’s disease dementia, Caregiving burden, Disease severity, Quality of life, Depression, Japan

## Abstract

**Background:**

Alzheimer’s disease (AD) dementia, a progressive neurodegenerative disease, exerts significant burden upon patients, caregivers, and healthcare systems globally. The current study investigated the associations between AD dementia patient disease severity and health-related quality of life (HRQoL) of both patients (proxy report) and their caregivers living in Japan, as well as caregiving-related comorbidities such as depression.

**Methods:**

This cross-sectional study used self-reported data from caregivers of people diagnosed with AD dementia by a healthcare provider in Japan. Caregivers were identified via online panels and invited to participate in an online survey between 2014 and 2015. Caregivers completed survey items for themselves, in addition to providing proxy measures for patients with AD dementia for whom they were caring. Patient and caregiver HRQoL was measured using the EuroQoL 5-Dimension (EQ-5D). Additional outcomes for caregivers of AD dementia patients included the Patient Health Questionnaire (PHQ-9) of depressive symptomology, as well as comorbidities experienced since initiating caregiving for their AD dementia patients. These outcomes were examined as a function of AD dementia severity, as measured by long-term care insurance (LTCI) categories. Bivariate analyses between LTCI and outcomes were conducted using independent t-tests and chi-square tests. Multivariable analyses, controlling for potential confounders, were conducted using generalized linear models (GLMs) specifying a normal distribution.

**Results:**

Across 300 caregiver respondents, multivariable results revealed that increasing AD dementia severity was significantly associated with poorer patient and caregiver EQ-5D scores and a high proportion of caregivers (30.0%) reported PHQ-9 scores indicative of major depressive disorder (MDD). The most frequent comorbidities experienced after becoming caregivers of AD dementia patients included hypertension (12.7%) and insomnia (11.0%). Depression and other comorbidities did not differ significantly by patient severity.

**Conclusions:**

The current study provides unique insight into the specific degree of incremental burden associated with increasing AD dementia severity among patients and caregivers alike. Importantly, greater disease severity was associated with poorer quality of life among both patients and caregivers. These results suggest that earlier detection and treatment of AD dementia may provide an opportunity to reduce the burden of disease for patients, caregivers, and society at large.

## Background

Alzheimer’s disease (AD) dementia is a progressive neurodegenerative disease that poses a significant burden to patients, caregivers, healthcare systems, and society. Disease symptomatology includes impaired cognition (e.g., memory difficulties) and is thus associated with difficulty in performing daily activities and consequent functional dependence on others for help. These symptoms and the progressive nature of AD dementia can result in increasing degrees of care required from formal (institutionalized/paid) and informal (family) caregivers [1]. The cause of AD dementia has not been fully identified and there is currently no cure; available treatments target cognitive symptoms and the management of behavioral symptoms [2].

Critically, the health, societal, and economic burden associated with AD dementia may increase as the severity of the disease increases [3–5], with both formal and informal AD dementia care contributing to this societal burden [6]. Disease severity has been found to be an independent predictor of time to institutionalized care and increased costs [7], while further studies have reported that increasing disease severity, including behavioral disturbances and greater symptomatology, is predictive of higher care costs [8–11]. A European/US based review reported that costs were more than doubled when comparing patients with severe dementia with those with mild dementia [12].

The high cost of informal care in AD dementia highlights the unique burden and responsibility placed on families of persons with AD dementia. Many AD dementia patients depend on care from family members due to the associated impairments in functioning and cognition that are hallmarks of this progressive disease. Notably, the care needs of patients with AD dementia can increase significantly as disease severity progresses [13], and studies have shown that family caregivers have a greater risk of medical illness, psychological impairment, disruption of personal and professional roles, and critically, an increased risk of mortality [3, 14–16].

Globally, the impact on caregivers has shown a range of negative outcomes in the context of AD dementia care [17, 18], including physical [15, 19], psychological [20, 21], social [22], and financial [23] consequences, with similar findings beginning to emerge in developing countries [18]. These findings have been documented in Japan, with research suggesting that family caregivers of elderly patients with dementia can experience significant burden and face various challenges associated with providing dementia-related care [24–31]. In a precursor to the present study, caregivers of individuals with AD dementia or other types of dementia in Japan were compared with matched non-caregivers in terms of their characteristics and health outcomes [32]. Consistent with international research, caregivers experienced significant impairments relative to their non-caregiver counterparts. Caregivers vs. non-caregivers reported poorer quality of life, lower levels of work productivity, and higher rates of mental health issues and healthcare utilization. Whereas AD dementia caregivers represent a vulnerable population at risk of negative health outcomes (especially as the disease progresses), evidence also suggests that some caregivers experience positive effects associated with caring for a family member diagnosed with a chronic or terminal disease, most often in the form of benefit-finding (e.g., strengthening of familial ties) or enhanced meaning [33–36].

Japan is ranked among the countries with the highest prevalence, with a 2010 study estimating 2.5 million adults suffering from dementia, a number predicted to reach 7.3 million by 2025 [37, 38]. To address the needs of care among a growing aging population, Japan implemented an insurance program and long-term care approach in 2000 that focused on consistent evaluation of the elderly and increased care and financial support for family caregivers [39]. AD dementia patients are majority users of long-term care insurance (LTCI) support for their care services. Despite this, there is notably little population-level empirical data concerning the specific impact of increasing disease severity on patient and caregiver outcomes, particularly assessing the relationship between these factors in the same study population. The current study provides important and novel insights into the incremental burden associated with increasing patient disease severity, and the relationship between burden and both patients’ and caregivers’ health outcomes. This further detailed quantification of burden, including among those with mild disease, can help provide valuable insight into the experience of patients and informal caregivers, guide decisions concerning burden and care needs, and help quantify the potential benefits/cost savings of effective management of this disease. The authors hypothesized that increasing patient disease severity would be associated with poorer patient and caregiver outcomes in Japan.

## Methods

### Sample

This cross-sectional study collected new data from caregivers of people with a clinical diagnosis of AD dementia who were previously identified in the 2012/2013 Japan National Health and Wellness Surveys (NHWS; internet-based questionnaires sampling adults aged 18 and older), with additional caregivers supplemented from Lightspeed Research (LSR) opt-in ailment panels. Convenience sampling, stratified by sex and age, was implemented to achieve demographic characteristics that match the adult population in Japan. Further details about the NHWS and respondent recruitment are provided in Laks et al. [40] and Goren et al. [32]. Inclusion criteria were: adults (18 years of age or older); caring for an individual diagnosed with AD dementia (per caregiver self-report); not receiving payment as a form of employment for caregiving duties; and providing informed consent. These new survey data were collected between December 2014 to January 2015. A sample of 300 caregivers was included in this analysis, including 119 current caregivers who were successfully recruited from the 2012 and 2013 NHWS and an additional 181 current caregivers who were recruited from the separate ailment panel source. Caregivers provided survey responses for themselves in addition to proxy measures on behalf of the AD dementia patient for whom they provided care.

### Measures

#### Sociodemographics and health characteristics

Sociodemographic variables included sex and age for both patients and caregivers. An additional background variable for patients included LTCI levels reported by their caregivers. The implementation of LTCI in Japan required detailed assessments of patients’ needs to effectively assign care resources. The Government-certified Disability Index (GCDI) is an 85-item measure developed to serve this purpose [41], providing a score from 0 to 5 that dictates the amount spent on services for a patient in each category and enables the severity of patient disease to be inferred. LTCI levels in the current study were trichotomized for analysis into categories reflecting increasing severity mapping roughly onto the GCDI levels: (1) low = no long-term nursing care insurance, support levels 1 or 2, or level not known; (2) medium = nursing care levels 1, 2, or 3; and (3) high = nursing care levels 4 or 5.

Additional background variables for caregivers included marital status, number of children in the household, change in employment status due to caregiving for the AD dementia patient, relationship with patient, and level of involvement with caregiving.

#### Patient disease severity

Patient disease severity was reported by caregivers based on patients’ LTCI scores. Previous research has demonstrated that LTCI is associated with other measures of disease severity [42], supporting its use as a proxy measure of AD dementia severity. Other measures that are associated with LTCI scores include the Short-Memory Questionnaire (SMQ) [43, 44] and an index of patient dependence.

#### Comorbidities

Charlson comorbidity index (CCI) [45] scores sum the weighted presence of comorbid conditions contributing to mortality risk. Higher scores indicate greater comorbid burden for the caregiver. Quan et al.’s [46] CCI scoring was implemented, as this updates the original algorithm on the basis of a more recent replication, with updated weighting and fewer conditions. Caregivers were asked if they had experienced these comorbidities since becoming caregivers of the AD dementia patients.

#### Health related quality of life

Two forms of the EuroQoL-5Dimension (EQ-5D) with 3 levels (Japanese version and value set), a widely used measure of health status, were used: one to assess the caregiver’s HRQoL, and a proxy version for the caregiver to rate the patient’s HRQoL from the patient’s perspective [47, 48]. The EQ-5D encompasses five dimensions (i.e., mobility, self-care, usual activities, pain/discomfort, and anxiety/depression), with responses corresponding to 3 levels of severity. The EQ-5D also generates a single health status index utility value ranging from 0 (death) to 1 (perfect health) [49, 50].

#### Depression

The Patient Health Questionnaire (PHQ)-9 [51, 52] was used to assess the frequency of caregivers’ depressive symptoms. A total score of 10 or higher [53] was used as an indicator of major depressive disorder (MDD). A “diagnosed depression” question was also included among other comorbidities experienced since initiating caregiving.

### Analysis

Descriptive analyses were conducted on all study variables of interest; these included *n*s and %s for categorical variables and means, medians, standard deviations (SDs), and minimum and maximum values for continuous variables. Bivariate associations were examined between the primary independent variable, LTCI, and several HRQoL and comorbidity dependent measures using chi-square tests for categorical variables (e.g., PHQ-9 MDD cut-off) and t-tests for continuous variables (i.e., EQ-5D), with two-tailed *p*-values indicating statistical significance. Results of these bivariate analyses helped inform variables that were included as covariates in multivariable analyses. Covariates were identified according to statistically significant differences in bivariate comparisons (*p* < .05) across the AD dementia severity categories. Some core variables of conceptual interest (e.g., age and gender) were included regardless of their association with the severity categories.

Multivariable analyses were used to assess patient and caregiver outcomes as a function of patient severity (i.e., burden as a function of LTCI levels, adjusting for potential measured confounders). The final list of identified covariates included: caregiver gender, caregiver age, caregiver employment status, caregiver marital status, percentage average daily care for which caregiver is responsible, percentage average daily care from visiting nurse/home-helper, total average hours of care required per day, years providing care for AD dementia patient, relationship between caregiver and patient, patient age, and patient gender. Separate generalized linear models (GLMs) specifying appropriate distributions (e.g., identity link function for normally distributed continuous outcomes, such as HRQoL) were chosen for each outcome of interest that was associated significantly with AD dementia severity in bivariate analyses. Multivariable models included exploratory interaction terms between age categories and employment status of caregivers on both caregiver and patient outcomes. These interaction terms were included to test whether the relationship between employment status and outcomes depended upon age of caregivers. These interaction terms were expected to account for cultural expectations surrounding age and work status (e.g., unemployment among younger adults may be associated with greater reductions in quality of life relative to older adults).

## Results

### Patient demographics

Across 299 patients (excluding 1 outlier *with a caregiver-reported patient age of 4 years*), the mean age was 83.7 years (*SD* = 7.6, median = 84), with minimum and maximum values of 38 and 106 years, and 78.7% of patients were female.

LTCI was used as a surrogate patient disease severity measure, with 12.3% of caregivers (*n* = 37 in this “low severity” group) indicating that they did not know their patients’ LTCI (*n* = 3) or that their patients had no LTCI (*n* = 15) or support level 1 (*n* = 7) or 2 (*n* = 12); 63.7% (*n* = 191 in this “medium severity” group) indicating that patients had nursing care level 1 (*n* = 52), 2 (*n* = 75), or 3 (*n* = 64); and 24.0% (*n* = 72 in this “high severity” group) indicating that patients had nursing care level 4 (*n* = 43) or 5 (*n* = 29) (see Table 1).

**Table 1:** Demographics and characteristics of patients (as reported by caregivers)

Characteristic		Total	
		%	*n*
Patient age (mean (SD), min to max)		83.70 (7.63)	38 to 106
Age category	< 80	23.7%	71
	80–84	30.3%	91
	85–89	23.7%	71
	90+	22.3%	67
Gender of patient	Female	78.7%	236
	Male	21.3%	64
Level of LTCI certification	No LTCI	5.0%	15
	Support level 1	2.3%	7
	Support level 2	4.0%	12
	Nursing care level 1	17.3%	52
	Nursing care level 2	25.0%	75
	Nursing care level 3	21.3%	64
	Nursing care level 4	14.3%	43
	Nursing care level 5	9.7%	29
	Don’t know	1.0%	3
Category of LTCI certification	Low severity	12.3%	37
	Medium severity	63.7%	191
	High Severity	24.0%	72

*Note*. *LTCI* long-term nursing care insurance, *SD* standard deviation

### Caregiver demographics

Across 300 caregivers (see Table 2), the average caregiver age was 53.9 years (*SD* = 11.0, median = 55), with minimum and maximum values of 20 and 96 years. A total of 61.0% were between the ages of 50 and 64 and 10.7% were 65 years of age or older. Of the caregiver sample, 55.0% were male, 62.3% were married or living with a partner, 51.0% had at least one dependent adult in the household, 17.7% had at least one dependent child in the household, and 26.7% reported changing employment status due to caregiving.

**Table 2:** Demographics and characteristics of AD dementia caregivers

Characteristic		Total	
		%	*n*
Caregiver age (mean (SD), min to max)		53.89 (11.02)	20 to 96
Age category	18–29	3.0%	9
	30–39	7.7%	23
	40–49	17.7%	53
	50–64	61.0%	183
	65+	10.7%	32
Gender of caregiver	Female	45.0%	135
	Male	55.0%	165
Marital status	Single	28.3%	85
	Married / living with partner	62.3%	187
	Divorced / separated / widowed	9.3%	28
Adults (18+) dependent in household	0	29.0%	87
	1	20.0%	60
	2	20.7%	62
	3	15.0%	45
	4+	15.3%	46
Children (< 18) dependent in household	0	82.3%	247
	1	8.7%	26
	2+	9.0%	27
Employed	Not employed, disabled, retired, student, or homemaker	31.0%	93
	Full-time, part-time, or self-employed	69.0%	207
Change in employment status due to caregiving for AD dementia patient	No	73.3%	220
	Yes	26.7%	80
Years providing care for the AD dementia patient	< 2 years	7.0%	21
	2 to < 4 years	33.7%	101
	4 to < 6 years	22.0%	66
	6 to < 10 years	17.3%	52
	10+ years	12.0%	36
	Can’t recall	8.0%	24
Caregiving role for AD dementia patient	Primary and only caregiver	30.3%	91
	Primary and sharing responsibilities with another person	30.0%	90
	Secondary caregiver	39.7%	119
Hours/week ‘personally responsible for providing care’	1–4 h	21.7%	65
	5–12 h	16.3%	49
	13–24 h	26.0%	78
	25–72 h (1–3 days)	20.0%	60
	73–168 h (3–7 days)	16.0%	48

*Note. AD* Alzheimer’s disease, *SD* standard deviation

Among those who recalled the total duration of caregiving (*n* = 276), 55.8% had provided at least 4 years of care; 13.0% had provided at least 10 years of care. Among all caregivers, 39.7% were secondary caregivers, 30.0% were primary caregivers but shared responsibilities, and 30.3% were the only primary caregivers. Caregivers were personally responsible for an average of 13.0 h (*SD* = 8.01) of care per day, with 48.0% of respondents responsible for 13 h of care per day or greater. Data were collected regarding the caregiver’s relationship to the patient (e.g., parent, spouse, offspring). Age and relationship data revealed that caregivers responded inconsistently regarding the direction of the parent vs. offspring relationship; therefore, responses were recoded into more logically defensible categories: offspring of patient (i.e., caregiver was at least 20 years younger than the patient), spouse/sibling/parent of patient (i.e., caregiver was older or no more than 20 years younger than the patient), or in-law/extended family/not related to patient (i.e., caregiver explicitly selected one of these options).

### HRQoL for caregivers and patients

**Fig. 1:**
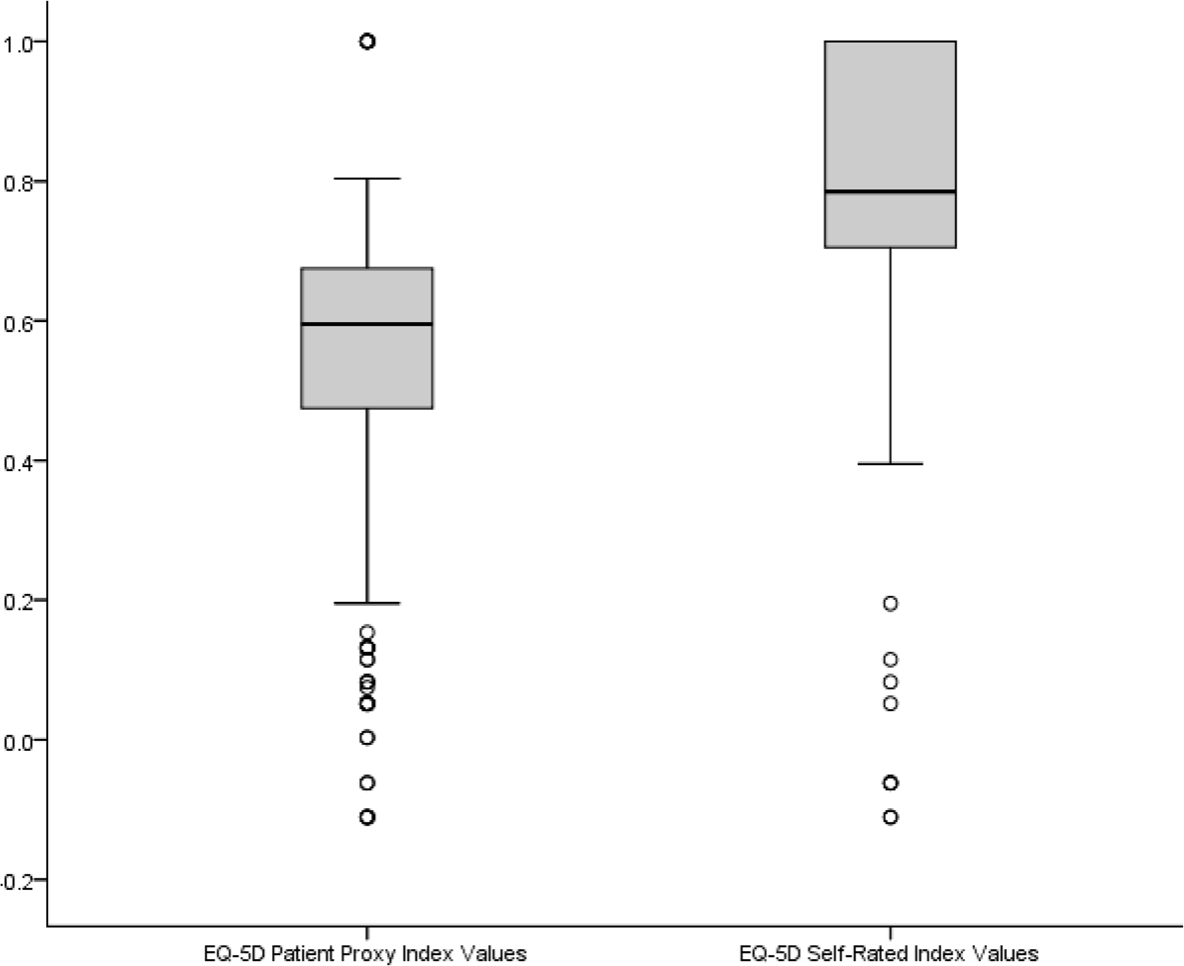
Box and whisker plot for EQ-5D patient proxy and caregiver values. *Note*: The ends of the boxes shown above depict the lower and upper quartiles while the horizontal line in the middle of each box depicts the median value. The whiskers extending from each box show the range of the lowest and highest observation. Outliers are identified with small circles (1.5× Interquartile Range)

EQ-5D patient proxy index values ranged from − 0.110 to 1.000, with a mean of 0.542 (*SD* = 0.224) and median of 0.595. EQ-5D caregiver index values ranged from − 0.110 to 1.000, with a mean score of 0.809 (SD = 0.216) and median of 0.785 (see Fig. 1).

The most common comorbidities that caregivers experienced since becoming caregivers included insomnia (11.0%) and hypertension (12.7%). Most caregivers (92.0%) reported no diagnosed comorbidities since becoming caregivers, however. A total of 90 re-contact caregivers (30.0%) experienced MDD as indicated via PHQ-9 scores ≥10 (see Table 3).

**Table 3:** Comorbidity of caregivers

Characteristic		Total	
		%	*n*
Depression (PHQ-9 scores)	None (0–4)	41.7%	125
	Mild depression (5–9)	28.3%	85
	Moderate depression (10–14)	13.0%	39
	Moderately severe depression (15–19)	10.0%	30
	Severe depression (20–27)	7.0%	21
MDD (PHQ-9 scores)	None (< 10)	70.0%	210
	MDD (10+)	30.0%	90
Diagnosed comorbidities	Depression	4.7%	14
	Insomnia	11.0%	33
	Anxiety	1.7%	5
	Hypertension	12.7%	38
	Pain	5.7%	17
	Diabetes (Type1/2)	1.0%	3
CCI experienced since caregiving	0	92.0%	276
	1	2.7%	8
	2+	5.3%	16

*Note. PHQ* Patient Health Questionnaire, *MDD* major depressive disorder

### Bivariate and multivariable results

**Fig. 2:**
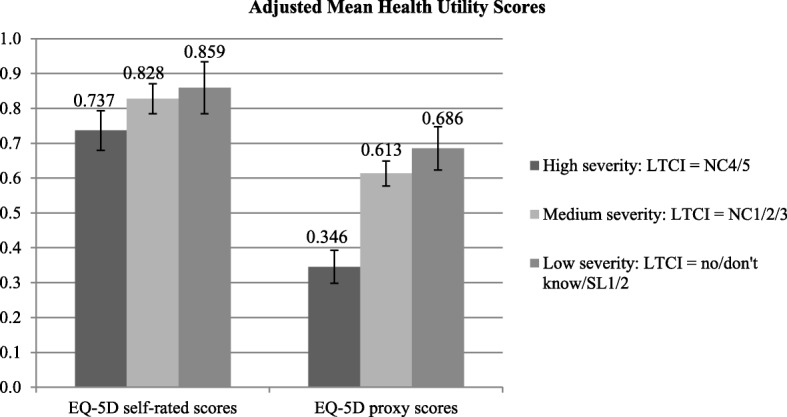
Adjusted means for health utility scores among AD dementia caregivers and patients. *Note.* EQ-5D scores are for AD dementia caregivers, EQ-5D proxy scores are for AD dementia patients as reported by their caregivers. *AD* Alzheimer’s disease, *LTCI* long-term care insurance, *NC* nursing care level, *SL* support level

Bivariate analyses demonstrated that AD dementia severity was associated with poorer patient HRQoL as measured by EQ-5D patient proxy scores. AD dementia patients at the low severity LTCI level experienced significantly and substantially higher EQ-5D proxy health utility scores (*M* = 0.69 *SD* = 0.16) than did those AD dementia patients at the medium severity LTCI level (*M* = 0.60, *SD* = 0.16) as well as patients at the high severity LTCI level (*M* = 0.31, *SD* = 0.24); *p*’s < .05. AD dementia severity significantly predicted EQ-5D scores for patients after adjusting for covariates in the multivariable model (see Table 4, Fig. 2).

**Table 4:** Results of generalized linear regression model of patient EQ-5D proxy index values as a function of LTCI & covariates (*n* = 300)

Characteristic	Reference Category	Variables	B	95% LCL	95% UCL	*P*-Value
(Intercept)			0.761	0.629	0.894	< 0.001
Patient characteristics						
AD dementia severity	Low severity	High severity	−0.340	− 0.414	− 0.267	< 0.001
		Medium severity	− 0.072	− 0.134	− 0.01	0.022
Sex	Female	Male	−0.030	− 0.078	0.018	0.220
Age	< 80 years	90+ years	0.001	− 0.067	0.069	0.974
		80–89 years	0.010	−0.045	0.064	0.728
Caregiver characteristics						
Sex	Female	Male	−0.041	− 0.085	0.002	0.064
Age X employment status	18–49 years and unemployed	65+ years and unemployed	0.026	−0.096	0.149	0.677
		50–64 years and unemployed	− 0.106	−0.202	− 0.009	0.032
	Unemployed and 18–49 years	Employed and 18–49 years	− 0.076	− 0.167	0.014	0.098
	65+ vs. 18–49 years and unemployed	65+ vs. 18–49 years and employed	0.026	−0.139	0.19	0.758
	50–64 vs. 18–49 years and unemployed	50–64 vs. 18–49 years and employed	0.144	0.038	0.249	0.008
% average daily care for which caregiver is responsible			0.000	0.000	0.001	0.378
% average daily care from visiting nurse/home-helper			−0.002	−0.003	0.000	0.008
Total average hours of care required per day			−0.042	−0.061	− 0.024	< 0.001
Years providing care for AD dementia patient	< 2 years	2 to < 4 years	0.022	− 0.061	0.105	0.603
		4 to < 6 years	0.050	−0.037	0.137	0.258
		6+ years	0.025	−0.062	0.112	0.575
		Can’t recall	0.084	−0.018	0.186	0.108
Marital status	Single	Married / living with partner	0.011	−0.037	0.059	0.660
		Divorced / separated / widowed	−0.068	−0.145	0.01	0.087
Relationship with patient	In-law, extended, not related	Offspring of patient (caregiver ≤20 years)	0.021	−0.031	0.074	0.420
		Spouse / sibling / parent of patient (caregiver > 20 years)	−0.048	−0.166	0.07	0.424
(Scale)			0.028	0.024	0.033	

*Note.* Presented are normal generalized linear model results. *AD* Alzheimer’s disease, *B* unstandardized beta, *LCL* lower confidence limit, *UCL* upper confidence limit

AD dementia severity was also associated with poorer caregiver quality of life as measured by EQ-5D scores. Caregivers of AD dementia patients at the high severity level of LTCI experienced significantly lower EQ-5D health utility scores (*M* = 0.75, *SD* = 0.28) than did caregivers of AD dementia patients at the low severity LTCI level (*M* = 0.84, *SD* = 0.17), *p* < .05. EQ-5D scores did not significantly differ between caregivers of patients at medium severity and low severity LTCI levels, however. After controlling for covariates, AD dementia severity remained a significant predictor of caregiver EQ-5D scores in the multivariable model (see Table 5). In both the EQ-5D patient and caregiver models, Age 50–64 X Employed interaction terms were statistically significant (see Table 4 and Table 5). These interaction terms indicate that although employment tended to be associated with poorer HRQoL for caregivers and patients, this difference was reduced among caregivers who were 50–64 years-old as well as patients with caregivers who were 50–64 years-old.

**Table 5:** Results of generalized linear regression model of caregiver EQ-5D self-rated index values as a function of LTCI & covariates (*n* = 300)

Characteristic	Reference Category	Variables	B	95% LCL	95% UCL	*P*-Value
(Intercept)			0.913	0.754	1.073	< 0.001
Patient characteristics						
AD dementia severity	Low severity	High severity	−0.122	−0.211	− 0.034	0.007
		Medium severity	−0.031	−0.105	0.043	0.414
Sex	Female	Male	0.003	−0.055	0.061	0.916
Age	< 80 years	90+ years	0.085	0.002	0.167	0.044
		80–89 years	0.019	−0.046	0.085	0.564
Caregiver characteristics						
Sex	Female	Male	−0.016	− 0.069	0.037	0.560
Age X employment status	18–49 years and unemployed	65+ years and unemployed	−0.014	−0.162	0.133	0.847
		50–64 years and unemployed	−0.081	−0.198	0.035	0.169
	Unemployed and 18–49 years	Employed and 18–49 years	−0.109	−0.217	0.000	0.050
	65+ vs. 18–49 years and unemployed	65+ vs. 18–49 years and employed	0.016	−0.182	0.214	0.871
	50–64 vs. 18–49 years and unemployed	50–64 vs. 18–49 years and employed	0.155	0.028	0.282	0.017
% average daily care for which caregiver is responsible			−0.001	−0.002	0.00	0.070
% average daily care from visiting nurse/home-helper			−0.002	−0.004	− 0.001	0.003
Total average hours of care required per day			−0.009	−0.032	0.013	0.424
Years providing care for AD dementia patient	< 2 years	2 to < 4 years	0.001	−0.098	0.100	0.984
		4 to < 6 years	0.030	−0.075	0.135	0.573
		6+ years	0.025	−0.079	0.130	0.634
		Can’t recall	0.002	−0.121	0.125	0.972
Marital status	Single	Married/living with partner	0.018	−0.040	0.076	0.535
		Divorced/separated/widowed	−0.021	−0.114	0.073	0.663
Relationship with patient	In-law, extended, not related	Offspring of patient (caregiver ≤20 years)	0.026	−0.036	0.089	0.411
		Spouse/sibling/parent of patient (caregiver > 20 years)	−0.022	−0.164	0.120	0.762
(Scale)			0.041	0.035	0.048	

*Note.* Presented are normal generalized linear model results. *AD* Alzheimer’s disease, *B* unstandardized beta, *LCL* lower confidence limit, *UCL* upper confidence limit

Among caregivers, AD dementia severity was not associated with developing any of the measured comorbidities since becoming caregivers, including anxiety, hypertension, and insomnia. AD dementia severity was also not associated with depression severity interval scores or MDD among caregivers as measured by the PHQ-9. PHQ-9 scores indicative of MDD among caregivers were, however, relatively high across AD dementia severity, including 35.1% of caregivers of low severity patients, 27.2% of caregivers of medium severity patients, and 34.7% of caregivers of high severity patients. Therefore, comorbidities were not explored further as outcomes in multivariable analysis.

## Discussion

The current study quantified the incremental burden associated with greater patient severity and its relationship with poorer patient and caregiver HRQoL. These findings reinforce the vulnerable nature of these two populations, both patients and caregivers, and the need for new interventions that can slow or halt disease progression and maintain or improve patient outcomes. These findings also emphasize the need for comprehensive supportive services for both patients and caregivers alike.

In previous studies, caregivers reported significant and broad impairment when compared with non-caregivers, even in the early stages of disease related to AD dementia or other types of dementia [32]. The current study extends these findings and suggests that severity specific to AD dementia is associated with greater burden among caregivers, encompassing impairments in both patients’ and caregivers’ HRQoL. Further, these results provide important and unique insights into the experience of patients and caregivers in the context of mild disease severity.

The current study is also consistent with past research that established that disease severity was associated with poorer patient and caregiver HRQoL [4, 5]. For example, Hessman and colleagues reported that increasing disease severity, as measured by the Mini-Mental State Examination (MMSE), was associated with significantly worse quality of life among patients in both institutional and community settings [5]. Studies have also suggested that caregivers’ quality of life can worsen over time [54], and a study by Kamiya and colleagues found that caring for patients with behavioral disturbances and lower scores on the MMSE (i.e., higher disease severity) was associated with increased caregiver burden [55]. A further study conducted in South Korea reported that more severe disease and greater functional impairment were also associated with increased caregiver burden [56], while a study by Ferrara and colleagues of 200 caregivers reported a proportional relationship between increased disease severity (as measured by MMSE) and caregiver burden (anxiety/depression). The authors suggested that disease severity was a primary force in the reorganization of the family unit as the level of care needed by the patient increased [57].

The current results, along with previous findings, reinforce the considerable burden associated with AD dementia, its progressive nature, and the increasing burden that can be associated with caregiving. This line of research emphasizes the need for programs or interventions (e.g., formal assistance programs) to be developed to support caregiving responsibilities and ultimately reduce burden and improve outcomes among patients and caregivers alike. For example, there is a cluster-randomized study that assessed psychosocial behavior management programme for home-dwelling people living with dementia [58]. This programme reduced challenging behaviors, which were evaluated by the Neuropsychiatric Inventory. This kind of intervention may also provide a host of benefits for patients, caregivers, and society. The potential to delay disease progression through early detection and prompt initiation of effective treatments/interventions have positive implications for patients’ and caregivers’ quality of life. Further research is needed to identify pharmacotherapies that can provide sustained relief from symptomatology and ideally modify the trajectory of the disease. Research on other types of interventions to help caregivers throughout the disease progression is also of high importance.

Importantly, a higher proportion of caregivers of AD dementia patients in this study reported depressive symptomology suggestive of MDD, compared with previous research that included caregivers of patients with dementia defined more broadly [32]. A total of 30.0% of the caregivers in this study reported PHQ-9 scores indicative of MDD, compared with 14.2% of caregivers in the previous study. These findings suggest that caring for AD dementia patients may be associated with greater burden relative to caring for patients with other types of dementia. Contrary to expectation, however, comorbid conditions such as MDD were not associated with severity of AD dementia, with high rates of depression reported among caregivers of those with both high and low severity disease. This suggests that caregivers may suffer from psychological distress even at the early stages of AD dementia, possibly due to the progressive nature of the disease and consideration of future care needs, and thus reinforcing the need for supportive care and treatment advances. Future studies should examine the relative burden of caregiving on HRQoL based on the type of dementia experienced by patients.

Although significant interactions were found between age (namely, 50–64-year-olds vs. 18–49-year-olds) and employment status, they did not reveal a consistent pattern across age groups. Speculatively, there was a non-significant trend toward poorer HRQoL with employment, suggesting that younger caregivers who were also employed (and potentially caring for children as well) may have tended to have an especially difficult time coping with the multiple demands of caregiving and maintaining a job, which may also have had an effect on patients’ HRQoL. This effect was reduced among 50–64-year-olds, perhaps implying that those with more established positions at work or without dependent children in the household were in relatively better shape to cope with the multiple demands.

### Study limitations

The online survey format of the current study provides certain sampling advantages but may under-represent caregivers without access to or sufficient comfort with online technology. This may result in a biased population from which surveys are drawn, such as respondents from higher socioeconomic backgrounds as well as respondents who are relatively young; however, it is important to note that if caregivers with access to and comfort with technology report burden associated with caregiving, the current study results may actually be an underestimation of burden among AD dementia caregivers in Japan.

There were a number of differences in demographic characteristics between the current study sample and the general population in Japan, with respondents in the current study being more likely to be younger in age (10.7% vs. 26.7% over age 65), male (55% vs. 48.7%), and socioeconomically advantaged (69% vs. 58.6% employed) [59]. These differences may reflect greater motivation, physical ability, and Internet access among an online-survey based study sample. Particularly of note is the high proportion of male caregivers, especially relative to the percentage of female patients (78.7%). Whereas the relatively large representation of male caregivers in this study contrasts with other similar international studies of AD dementia caregivers, in which female caregivers were the majority [60], this study may provide unique insight into a group that is typically underrepresented in this field of study.

A further notable limitation of the current study is the cross-sectional, contemporaneous nature of the data, which precludes causal assessment (e.g., the change in burden with transition across patient disease states within caregivers). For example, greater caregiver burden may lead to the implementation of increasing levels of nursing care, thus resulting in a positive correlation between these two factors. Further research is needed to examine these relationships over time and to best guide future policy and supportive care initiatives.

The current study also utilized a proxy measure of patient HRQoL completed by their caregivers. Whereas evidence suggests this to be appropriate [61], previous studies have shown that discrepancies can exist in the ratings of patient outcomes based on the rater (patient versus caregiver). Whereas spouses or partners who live with the patient have been found to provide the most reliable informant ratings [62], a Taiwan-based study reported that such discrepancies could be associated with the quality of the patient-caregiver relationship, with greater discrepancies reported between dyads with poorer relationships on an HRQoL measure [63]. In a two-year study of 574 AD dementia patients and their caregivers, researchers found that while patient-reported HRQoL was stable over the course of the study, disease severity markers and caregiver-rated QoL declined [64]. Given these possible influencing factors, caution is warranted in interpreting proxy-based assessment of patient outcomes.

## Conclusions

The current findings provide specific quantification of burden among AD dementia patients and caregivers alike, and suggest that greater AD dementia severity is associated with a higher degree of burden, most notably related to HRQoL. This study also provides important and novel insight into the experience of those with mild disease symptoms, a group that has received little attention in previous research. In the context of an aging global population and the progressive nature of AD dementia, these results further highlight the need for efforts in promoting early detection and treatment, an issue that has received increasing attention through national organizations in Japan and globally [65, 66]. New therapies that promise to slow or halt AD dementia progression (once it has been diagnosed), and thus maintain patients in a milder disease state, are needed. The benefits of early detection and treatment could provide a significant reduction in disease-related burden for patients, caregivers, and society at large.

## Abbreviations

AD: Alzheimer’s disease
CCI: Charlson comorbidity index
EQ-5D: EuroQol-5Dimension
GCDI: Government-certified Disability Index
HRQoL: Health-related quality of life
LTCI: Long-term nursing care insurance
MDD: Major depressive disorder
MMSE: Mini-Mental State Examination
NHWS: National Health and Wellness Survey
PHQ: Patient Health Questionnaire
SD: Standard deviation
SMQ: Short-Memory Questionnaire
